# Core–envelope miscibility in sub-Neptunes and super-Earths

**DOI:** 10.1038/s41586-025-09970-4

**Published:** 2026-01-21

**Authors:** Travis Gilmore, Lars Stixrude

**Affiliations:** Department of Earth, Planetary, and Space Sciences, University of California, Los Angeles, Los Angeles, CA USA

**Keywords:** Exoplanets, Core processes

## Abstract

Sub-Neptunes and super-Earths, the most abundant types of planet in the galaxy, are unlike anything in the Solar System, with radii between those of Earth and Neptune^1,2^. Fundamental questions remain regarding their structure and origin. Although super-Earths have a rocky composition^3^, sub-Neptunes form a distinct population at larger radii and are thought to consist of a rocky core overlain by a hydrogen-rich envelope^4,5^. At the extreme conditions of the core–envelope interface (exceeding several gigapascals and several thousand kelvin^4,6^), reaction between core and envelope seems possible, but the nature and extent of these reactions are unknown. Here we use first-principles molecular dynamics driven by density functional theory to show that silicate and hydrogen are completely miscible over a wide range of plausible core–envelope pressure–temperature conditions. We find the origin of miscibility in extensive chemical reaction between hydrogen and silicate, producing silane, SiO and water species, which may be observable with ongoing or future missions. Core–envelope miscibility profoundly affects the evolution of sub-Neptunes and super-Earths, by dissolving a large fraction of the hydrogen of the planet in the core and driving exchange of hydrogen between core and envelope as the planet evolves.

## Abstract (web summary)

First-principles molecular dynamics driven by density functional theory is used to show that silicate and hydrogen are completely miscible over a wide range of plausible core–envelope pressure–temperature conditions.

## Main

Both sub-Neptunes and super-Earths may have begun with a hydrogen-rich envelope gravitationally captured from the surrounding stellar disk during accretion. Sub-Neptunes retained some portion of this primary atmosphere to the present day, whereas in the case of super-Earths, the envelope was subsequently lost^7^. The population of super-Earths and sub-Neptunes, and the origin of the radius valley that separates these two classes of planets, is best explained by cores that are made of an Earth-like composition without a substantial amount of accreted ice^8–11^. For sub-Neptunes, the hydrogen-rich envelope overlies the rocky core for billions of years, whereas for super-Earths, the envelope may be retained for about 100 Myr (refs. ^2,8,12^). During this time, the hydrogen envelope and the rocky core may have reacted chemically with each other, altering the composition of the atmosphere and interior. Our own Earth may have also once had a primary atmosphere that left its mark on the present-day composition of its interior^13,14^.

The structure and evolution of sub-Neptunes and super-Earths challenge our understanding of fundamental properties of materials at extreme conditions. For example^6^, at the core–envelope boundary of a typical sub-Neptune, the temperature may be 5,000 K, whereas the pressure may be 5 GPa. Both core and envelope are fluid at the conditions of the interface, which exceed the silicate melting temperature: the rocky core forms a magma ocean^6,15,16^. This part of the pressure–temperature space is not much explored by experiments or theory. Similar temperatures are found in the interiors of giant planets, but only at much higher pressures, at which dynamic compression experiments are able to probe^17^. By the same token, similar pressures are found in the interiors of rocky planets, but only at much lower temperature conditions that are readily accessible to static compression experiments^18^. Previous modelling studies have emphasized the importance of a better understanding of fluid silicate–hydrogen phase equilibria for super-Earth and sub-Neptune structure and evolution^19^. Experiments have demonstrated that small concentrations of hydrogen are soluble in silicate liquid, albeit at pressure–temperature conditions much less extreme than those we consider here^18^.

**Fig. 1:**
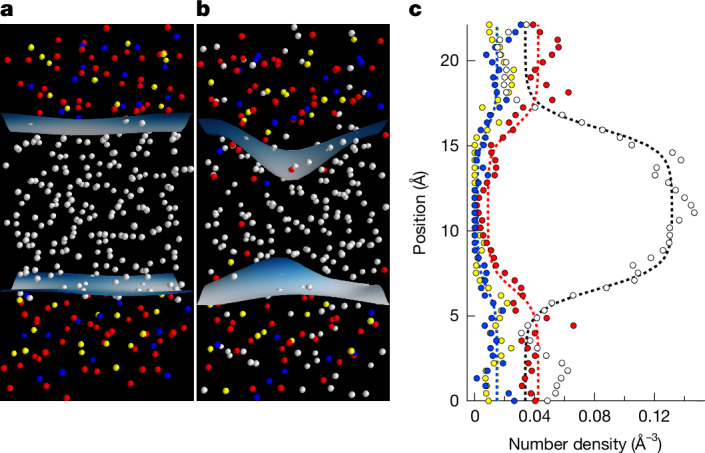
Illustration of the phase coexistence method. **a**,**b**, Snapshots from a phase coexistence simulation at 10 GPa and 2,500 K: the initial condition (**a**) and from the equilibrated portion of the trajectory (**b**). H, white; Mg, yellow; Si, blue; O, red; and Gibbs dividing surfaces, light blue. **c**, The one-dimensional density profile from the equilibrated portion of the trajectory (symbols with colours corresponding to atom types); lines are fits to equation (4).

We use the phase coexistence method^20,21^ to study the reaction between hydrogen fluid and a liquid of MgSiO_3_ composition, taken to be representative of the rocky portion of planets (Fig. 1 and Methods). The initial condition consists of equal-sized domains of pure hydrogen and pure silicate composition, pre-equilibrated to the same pressure and temperature. The Hellmann–Feynman forces are computed in density functional theory. As the molecular dynamics simulation proceeds, a reaction occurs, and a dynamic equilibrium is established. At temperatures below the critical temperature, two phases with distinct compositions coexist in equilibrium. By quantifying the compositions of the two coexisting phases, we map out the phase diagram and identify the critical temperature above which a single homogeneous fluid is stable. To gain additional insight into our results, we also perform a series of simulations of homogeneous fluids with compositions on the MgSiO_3_–H_2_ join, from which we extract the energetics of solution. Further details are given in the Methods.

Over a range of conditions, we find two phases coexisting in dynamic equilibrium, one hydrogen-rich and the other hydrogen-poor (Fig. 1). In both phases, the silicate fraction nearly maintains MgSiO_3_ stoichiometry, that is, MgSiO_3_ behaves as a component, and the system is nearly binary. We choose as our compositional variable the molar fraction of the H_2_ component

1 $$x=\frac{{N}_{{\mathrm{H}}_{2}}}{{N}_{{\mathrm{H}}_{2}}+{N}_{\mathrm{MgO}}+{N}_{{\mathrm{SiO}}_{2}}}=\frac{{N}_{\mathrm{H}}/2}{{N}_{\mathrm{H}}/2+2({N}_{\mathrm{Mg}}+{N}_{\mathrm{Si}}+{N}_{\mathrm{O}})/5}$$

where *N*_*i*_ is the number of molecules or atoms of type *i*. We quantify the composition of the two coexisting phases based on (1) one-dimensional density profiles using a Widom-like expression^22^ and (2) a three-dimensional coarse-grained density field^23^ (see Methods for further details).

**Fig. 2:**
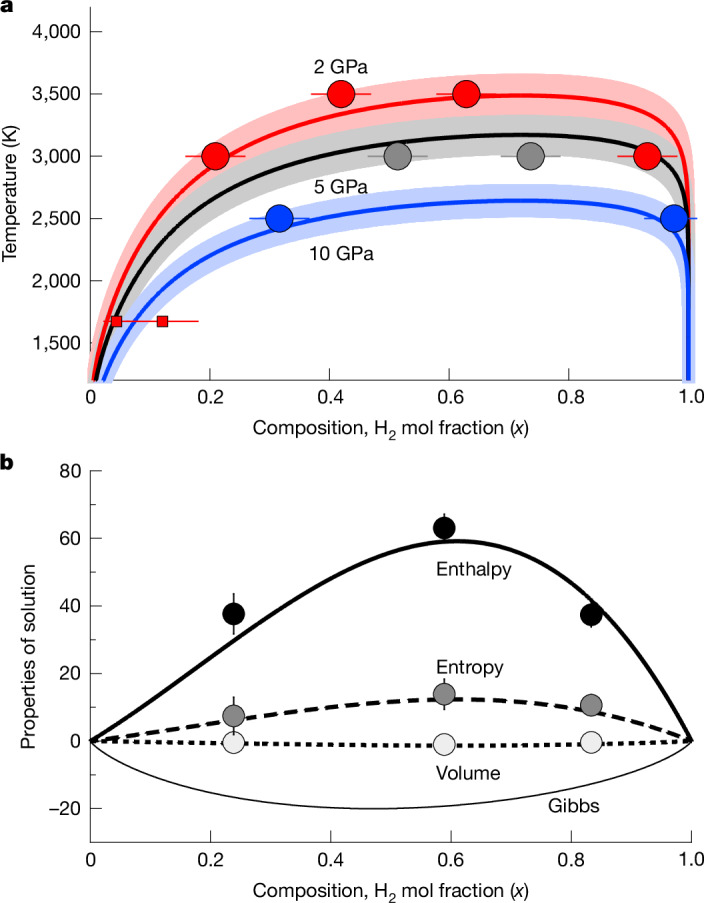
Phase diagram and energetics of solution. **a**, Composition of the two coexisting phases from our simulations (symbols) near 2 GPa (red), 5 GPa (grey) and 10 GPa (blue) with uncertainties in composition indicated (Extended Data Table 1). Curves with corresponding colours are our computed phase equilibria with estimated uncertainty indicated by shading. Two experimental measurements of the hydrogen solubility in mafic melt^18^ at 2 GPa (red squares with error bars) are also shown. **b**, Excess properties of solution at 5 GPa and 4,000 K from our homogeneous simulations: enthalpy (kJ mol^−1^) (solid line and black symbols), entropy (J mol^−1^ K^−1^) (long dashed line and dark grey symbols) and volume (cm^3^ mol^−1^) (short dashed line and light grey symbols). The total Gibbs free energy of solution (thin solid line) is also shown. Curves in **a** and **b** are computed from the same asymmetric regular solution model (Methods).

The compositions of the two coexisting phases approach each other with increasing temperature (Fig. 2a and Extended Data Table 1) and merge into a single homogeneous phase at temperatures greater than the critical temperature *T*_C_. We find that the compositions of the two phases are asymmetric: the hydrogen-poor phase has a greater amount of hydrogen than the amount of silicate in the hydrogen-rich phase. An asymmetric regular solution model captures our results (see Methods for further details).

**Fig. 3:**
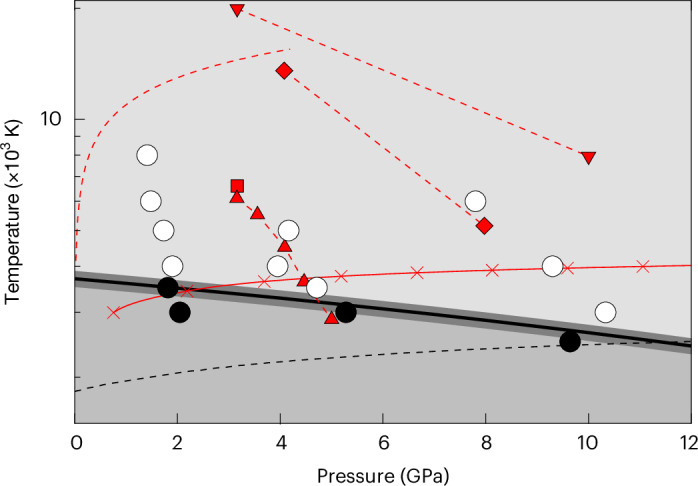
Phase diagram and core–envelope interface conditions. The equilibrated conditions of our simulations: black circles represent the conditions at which two phases coexist; white circles represent the conditions at which a single homogeneous phase is stable (uncertainties in pressure and temperature are smaller than the size of the symbols). The black line is the critical curve *T*_C_(*P*) and is computed from the same asymmetric regular solution model used to generate the curves in Fig. 2 (see Methods); the shading provides an estimate of the uncertainty in *T*_C_. The black dashed line is the MgSiO_3_ liquidus^59^. Our results are compared with the conditions at the core–envelope interface in models of sub-Neptunes from the literature (red symbols and dashed lines): squares^29^ refer to rocky planets accreting by pebbles from 0.75*M*_⊕_ to 2.5*M*_⊕_ within a stellar disk at 1 au; down triangles^15^ represent Kepler-36c, outer core–envelope boundary at the beginning of the isolation stage (hotter) and at 7 Gyr; diamonds^36^ represent a sub-Neptune with *M* = 4*M*_⊕_ at the end of the accretion stage (hotter) and at the end of the mass loss stage when the envelope mass *M*_env_/*M* = 0.025; red dashed line with no symbols^30^ represents rocky planets embedded in the disk and accreting by planetesimal impact from 0.1*M*_⊕_ (colder) to 3.8*M*_⊕_; up triangles^16^ denote a sub-Neptune with mass *M* = 4*M*_⊕_ and hydrogen envelope mass *M*_env_/*M* = 0.02 on cooling from 1 Myr (hotter) to 10 Gyr in decade increments. The red solid line shows the conditions at the core–envelope interface in sub-Neptunes with *M* = 1–8*M*_⊕_, *M*_env_/*M* = 0.02, equilibrium temperature *T*_eq_ = 500 K and intrinsic temperature *T*_int_ = 70 K, with the crosses marking unit increments in *M*/*M*_⊕_, following existing methodology^16,36^.

Homogeneous simulations show that immiscibility between hydrogen-poor and hydrogen-rich fluids originates in a positive enthalpy of solution (Fig. 2b and Extended Data Table 2). The excess entropy of solution far exceeds the ideal entropy of mixing (5.8 J mol^−1^ K^−1^), producing solvus curves (binodes) that are flat-topped: that is, the compositions of the two coexisting phases diverge rapidly from each other on cooling below *T*_C_. The negative volume of solution causes *T*_C_ to decrease with increasing pressure: from 3,500 K at 2 GPa to 2,600 K at 10 GPa (Fig. 3).

The behaviour that we find was not anticipated by previous studies. Estimates of the solubility of hydrogen in silicate melt^24–27^ used an expression based on the solubility of noble gases (Henry’s law)^28^. This expression cannot capture complete miscibility because it assumes that hydrogen is inert. For example, one parameterization^24^ yields 20 mol% solubility of H_2_ in silicate melt at 2 GPa, 4,000 K, and the other two studies yield smaller solubilities, as compared with the unlimited solubility (that is, miscibility) that we find. Estimates of the solubility of silicate in hydrogen fluid^15,29–32^ assumed that silicate solubility is limited by congruent silicate vaporization and ideal mixing, leading to underestimated solubility: for example, 0.2 mol% of silicate in hydrogen at 2 GPa, 4,000 K, as compared with the unlimited solubility that we find. A previous study^19^ argued that *T*_C_ should be greater than or equal to the congruent vapour–liquid critical temperature *T*_silicate_, and that by analogy with the water–hydrogen system, *T*_C_ might increase with increasing pressure. However, we find *T*_C_ (3,500 K at 2 GPa) to be substantially less than *T*_silicate_ (6,600 K at 0.14 GPa) (ref. ^20^) and *T*_C_ decreasing with increasing pressure, illustrating the importance of fully non-ideal interactions as in our simulations. Complete miscibility in the fluid silicate–water system is well known^33–35^, and partly by analogy, a recent study speculated that miscibility might occur in the silicate–hydrogen system as well^36^. However, the silicate–water system is fundamentally different as reaction does not require redox exchange, unlike the silicate–hydrogen system.

**Fig. 4:**
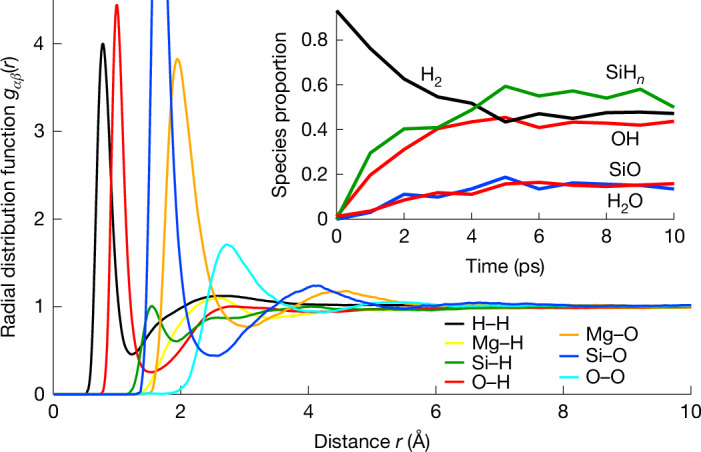
Speciation in the miscible fluid. Evolution of the speciation in a phase coexistence simulation at 5 GPa and 4,000 K that ultimately becomes homogeneous (inset) and the structure of the equilibrated homogeneous fluid (main figure). The main figure shows the H– and O– partial radial distribution functions and the inset shows the proportions of H atoms that are bound to one other H atom (H_2_) (black), the proportion of O atoms that are bound to one (OH) or two H (H_2_O) atoms (red), and the proportion of Si atoms that are bound to a single O atom (SiO) (blue), or to any number of H atoms (SiH_*n*_) (green).

We probe the microscopic mechanism of reaction between the two phases by examining the evolution of species during equilibration (Fig. 4). Initially, phase coexistence simulations have all H as H_2_, all O bonded to Si and Si surrounded by four O on average with no SiO. As the simulation proceeds (towards a homogeneous state in the example shown), H_2_ diminishes at the expense of hydroxyl and molecular water species, and SiO and SiH_*n*_ increase in abundance. These changes are captured by the redox reactions

2 $${\mathrm{SiO}}_{2}+{\mathrm{H}}_{2}\to \mathrm{SiO}+{\mathrm{H}}_{2}\mathrm{O}$$

3 $${\mathrm{SiO}}_{2}+3{\mathrm{H}}_{2}\to {\mathrm{SiH}}_{4}+2\mathrm{OH}$$

producing reduced Si species SiO and SiH_4_, while oxidizing H. We confirm the presence of O–H and Si–H bonding in our simulations by computing the radial distribution function^37^ over the equilibrated, homogeneous portion of the simulation, which shows clear peaks at short distance (<2 Å) in the O–H and Si–H radial distribution functions (Fig. 4). For illustration, we have written equation (3) in terms of SiH_4_, although in the simulation shown in Fig. 4 SiH_4_ is subordinate to lower coordinate species SiH_*n*_ (*n* < 4). However, we find that silane becomes more abundant than lower coordinate SiH_*n*_ species at greater hydrogen concentration, for example, in the hydrogen-rich fluid in phase coexistence simulations performed at temperatures less than *T*_C_. Trace amounts of silane and water have been identified as reaction products in experiments in the MgSiO_3_–H_2_ system, albeit at much lower temperatures than our simulations and without the ability to quantify abundances^38^. Apart from the redox reactions, the relative amounts of OH and H_2_O are governed by H_2_O + O → 2OH, where the O is bonded to Si (ref. ^39^).

The products of reactions (2) and (3) may be observable by the James Webb Space Telescope (JWST). H_2_O has been observed in the atmosphere of sub-Neptunes at concentrations greater than stellar metallicity^5,40–42^. Determining whether the observed water enrichment is endogenous, that is, due to core–envelope reactions, such as equation (2), or from accreted ice from beyond the snow line^43–45^, is central to understanding the origin of these planets^46^. Some previous planetary modelling studies have proposed that water is produced primarily by reaction of hydrogen with FeO (ref. ^26^), but reaction of hydrogen with SiO_2_ (equation (2)) may be a more important source of water^27^ because SiO_2_ is expected to be much more abundant in rocky cores than FeO (ref. ^47^). Previous studies have assumed ideal mixing between species from the low pressures to which observations are most sensitive (10^−6^ GPa) all the way down to the core–envelope boundary (5 GPa) (refs. ^26,27,48^), and it will be important in future to include the strongly non-ideal effects that we find to evaluate whether some or all of the observed water enrichment is endogenous. Silicon species have not yet been identified in the atmospheres of sub-Neptunes, but the possibility of observing SiO with JWST has been evaluated in detail^49^, and silane has been observed in a brown dwarf with JWST^50^. Planetary modelling studies have examined whether core–envelope reactions such as equations (2) and (3) can produce silicon species at detectable levels high in the atmosphere^27,32,51,52^. Among the important considerations for future studies will be to include the non-ideal thermodynamics that we find and the incongruent saturation and condensation of silicon species.

Our results indicate that the miscibility of core and envelope must be considered in models of the structure and thermal evolution of sub-Neptunes (Fig. 3). The reason is that probable pressure–temperature conditions at the core–envelope boundary lie well above the critical curve, in the regime of complete miscibility between silicate and hydrogen. For example, in a thermal evolution model of a sub-Neptune that assumes no reaction between core and envelope, the conditions at the core–envelope boundary lie above the critical curve throughout the first billion years of evolution (for example, 4.5 GPa, 3,600 K at 1 Gyr) (ref. ^16^). This model is, therefore, not in chemical equilibrium. Earlier states of evolution are likely to be even hotter and lie even farther above the critical curve. For example, the core–envelope boundary lies at 4 GPa and 13,000 K in a recent model at the end of the accretion phase^36^, and even hotter conditions are found during accretion^15,30^. Although the precise conditions at the core–envelope boundary depend on many factors, including the mass of the rocky core, how much gas the core accretes and how much it retains after disk dispersal, the pressure–temperature conditions that we have highlighted are typical of a wide variety of sub-Neptune models at various stages of evolution^6,16^. The only sub-Neptunes that do not exhibit H_2_–MgSiO_3_ miscibility are less massive (<4*M*_⊕_), older (≥10 Gyr) or with lower envelope mass fractions (<0.02) than most of the sub-Neptune population.

Our results have important implications for every stage of the evolution of sub-Neptunes, from the accretion phase to boil off, to present-day observations of atmospheric chemistry. Consider first the accretion phase in which a sub-Neptune forms by accreting a rocky core while embedded in the disk^6,53^. The rocky core gravitationally captures a portion of the disk, forming a hydrogen-rich envelope. Previous analyses of this stage assume limited dissolution of hydrogen in the core, or silicate dissolution in the envelope dictated by the congruent vapour pressure of the silicate, while neglecting hydrogen solubility in the silicate^15,24,25,29,30,36^. Our results indicate that these are not good assumptions. If the planet maintains chemical equilibrium at the interface between the envelope and the core, then silicate and hydrogen are completely miscible over a wide range of plausible evolutionary scenarios (Fig. 3).

As the disk disperses, the planet loses mass by evaporation. If the envelope and core are chemically distinct, most of the envelope mass is lost during this phase: for example, in one scenario^54^ 85% of the envelope is lost to boil off. However, if much of the hydrogen envelope is dissolved in the core, then a greater fraction of hydrogen may be retained by the planet after disk dispersal. A more in-depth analysis of this problem would consider the complete miscibility that we find, along with variations of the pressure–temperature conditions at the core–envelope interface that occur as a result of boil-off, and the attendant changes in the phase equilibria.

After boil-off, the planet cools, altering the pressure and temperature conditions at the core–envelope interface. As the equilibrium between hydrogen-rich and hydrogen-poor phases evolves on cooling, partitioning of hydrogen between the core and envelope alters, leading to changes in the mass of the envelope and the planetary radius. Some sub-Neptunes may cross from earlier hotter states that are super-critical to later colder states that are sub-critical (Fig. 3), leading to changes in the composition of core and envelope that may involve silicate condensation, rain-out^55^, cloud formation or regions of convective inhibition^19,56^. Some sub-Neptunes may lose their envelopes because of photo-evaporation or core-powered mass loss, forming super-Earths^8,9^. The formation of super-Earths may also, therefore, be affected by silicate–hydrogen miscibility: if much of the hydrogen of sub-Neptunes is dissolved in the rocky core, the hydrogen may be less susceptible to loss^24^. Some super-Earths may have small low-molecular-weight atmospheres that originate in core–envelope reactions^57^, and even those that have completely lost their envelopes may still bear the imprint of chemical reaction between core and the envelope, including sequestration of hydrogen to a metallic core, and the production of endogenous water^14,58^.

## Methods

### Phase coexistence method simulations

Our simulations follow the phase coexistence method described in our previous work^20,21,60^. First-principles molecular dynamics simulations are based on density functional theory in the PBEsol approximation^61^ using the projector augmented wave method as implemented in VASP (Vienna Ab initio Simulation Package)^62,63^. We assume thermal equilibrium between ions and electrons using the Mermin functional^64^. Sampling the Brillouin zone at the Gamma point and a basis set energy cutoff of 500 eV converges the total energy and pressure to within 3 meV per atom and 0.2 GPa, respectively. The outermost core radii (in Bohr) and number of electrons treated as valence are as follows: H (1.1,1), Mg (2.0,2), Si (1.9,4) and O (1.52,4); the simulations are non-spin polarized.

We perform phase coexistence simulations in the canonical ensemble with a Nosé–Hoover thermostat^65,66^ with a time step of 0.1–1.0 fs for 10–70 ps, sufficient to achieve equilibration, and with drift of the conserved quantity not exceeding 1.5 meV atom^−1^ ps^−1^. For the longest time steps (1.0 fs), we used a simple form of hydrogen mass rescaling^67^, setting the mass of the hydrogen atom to 4 u or 16 u. We prepare the initial condition by first performing a homogeneous simulation of MgSiO_3_ fluid at temperature *T* in a cubic simulation cell containing 135 atoms, adjusting the dimension *L* until the pressure *P* matches the desired pressure (2 GPa, 5 GPa or 10 GPa). We then perform a homogeneous simulation of H fluid at *T* in a simulation cell of identical dimensions and adjust the number of H atoms until the pressure *P* matches that of the MgSiO_3_ simulation. The number of H atoms, therefore, varies depending on *P* and *T*, reflecting the contrast in compressibility and thermal expansivity between MgSiO_3_ and H fluid. We form the initial configuration of the phase coexistence simulation by combining equilibrated snapshots of MgSiO_3_ and H fluid, forming a simulation cell of dimensions *L* × *L* × 2*L*.

**Extended Data Fig. 1:**
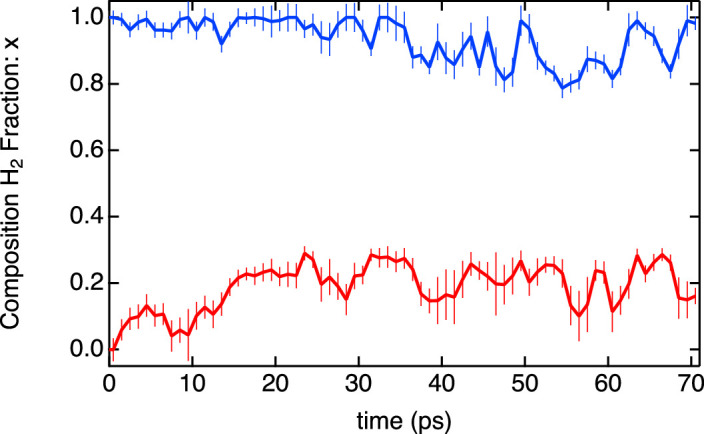
Phase coexistence equilibration. Equilibration of the compositions of the hydrogen-rich phase (blue) and the hydrogen-poor phase (red) versus time in the phase coexistence simulation at 2 GPa and 3000 K. Phase composition determinations are shown every 1 ps with uncertainties derived from the fit to equation (4).

We quantify the compositions of the two coexisting fluids in two ways^20,21^. First, we use the one-dimensional density profile normal to the interface, which we find follows the expected hyperbolic tangent form^22^

4 $$\rho (z)={\rho }_{2}+\frac{{\rho }_{1}-{\rho }_{2}}{2}\mathop{\sum }\limits_{j=1}^{2}{(-1)}^{j}\tanh \frac{(z-{z}_{1})-{\rm{nint}}(z-{z}_{1})+{(-1)}^{j}w}{\delta }$$

where *ρ*_1_ is the number density of the atom type (H, Mg, Si or O) in the phase with its centre of mass located at the scaled coordinate *z*_1_ and half-width *w*, *ρ*_2_ is the number density of the atom type in the other phase, *δ* is the interface width, the nint function accounts for periodic boundary conditions, and the sum accounts for the presence of two interfaces. An example of a fit of this form to our results is shown in Fig. 1. An example of the equilibration of the phase compositions as a function of time is shown in Extended Data Fig. 1.

Furthermore, we calculate^23^ the interface between the two phases as the iso-density surface **s** with density *c*

5 $$\overline{\rho }({\bf{s}},t)=c$$

and the coarse-grained three-dimensional density field

6 $$\bar{\rho }({\bf{r}},t)=\sum _{i}{(2{\rm{\pi }}{\xi }^{2})}^{-\frac{3}{2}}\exp \left[-\frac{{({\bf{r}}-{{\bf{r}}}_{i})}^{2}}{2{\xi }^{2}}\right]$$

where **r** is the position vector, **r**_*i*_ is the position of atom *i*, and *ξ* is the coarse-graining length, which we take to be 2.5 Å. The interface remains quasi-planar throughout the course of the simulation, with the magnitude of the fluctuations of the interface related to the surface tension^68^ (Fig. 1). The time-averaged interface is planar and represents the Gibbs dividing surface.

### Homogeneous simulations

Apart from phase coexistence simulations, we also perform homogeneous simulations in the *NPT* ensemble, with a Parinello–Rahman barostat^69,70^, a Langevin thermostat^71–73^ and a time step of 0.1 fs for a duration of 10 ps. We perform all homogeneous simulations at 5 GPa and 4,000 K with varying compositions across the MgSiO_3_–H_2_ join and 135–160 total atoms in equant simulation cells. These *NPT* simulations yield the enthalpy and the volume. For the entropy, we use the two-phase thermodynamic-memory function (2PT-MF) method^74,75^, which decomposes the vibrational density of states into solid-like and fluid-like portions. As in our previous phase coexistence study^21^, we found that *NPT* simulations were not appropriate for the determination of the entropy because the Langevin thermostat biases the vibrational density of states. To compute the entropy, we therefore continued our *NPT* simulations in the canonical ensemble at the time-averaged equilibrium volume for another 10 ps. Uncertainties are computed using the blocking method^76^.

### Compositional model

**Extended Data Fig. 2:**
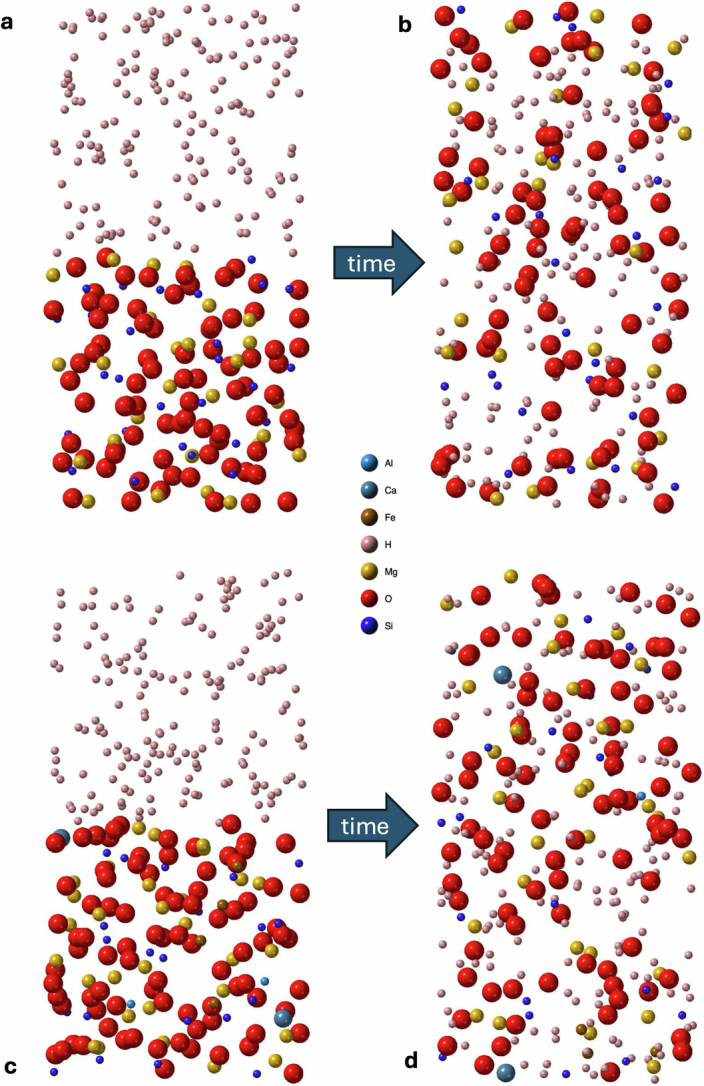
Influence of silicate composition. Snapshots from simulations in H_2_-silicate systems: (**a,b**) the silicate composition is MgSiO_3_ with *N*_Mg_ = 27, *N*_O_ = 81, *N*_Si_ = 27, and *N*_H_ = 160; (**c,d**) a silicate composition taken to be that of the bulk silicate Earth as used by us in our simulations of the terrestrial magma ocean^77^ with *N*_Al_ = 2, *N*_Ca_ = 2, *N*_Fe_ = 4, *N*_Mg_ = 30, *N*_O_ = 87, *N*_Si_ = 24, and *N*_H_ = 170. On the left (**a,c**) are the initial snapshots and on the right (**b,d**) are snapshots from the equilibrated portion of the simulation at 10 ps.

We have taken MgSiO_3_ composition to be representative of the rocky portions of planets, as MgO and SiO_2_ are expected to be the main oxides, making up, for example, 90% of the bulk silicate Earth^47^. To test whether other oxide components may substantially alter the critical temperature that we find, we have performed an additional phase coexistence method simulation in which the silicate composition is a more accurate model of the bulk silicate Earth as used by us recently in our study of the terrestrial magma ocean^77^. The outermost core radii and number of valence electrons of the additional elements are Al (1.9,3), Ca (3,8) and Fe (2.2,14). Otherwise, the simulation setup is the same as for the H_2_–MgSiO_3_ system. The results indicate that the richer silicate system also produces complete miscibility at 5 GPa, 4,000 K (Extended Data Fig. 2). This shows that a range of plausible silicate compositions is likely to produce complete miscibility at similar temperatures to what we find in the MgSiO_3_–H_2_ system.

Other elements that we have not considered are also unlikely to substantially affect the critical temperature because they are likely to occur in small concentrations in sub-Neptunes and super-Earths. For example, replacing H_2_ with a gas of solar composition^78^ in our phase coexistence method simulations, leads to 14, 0.04 and 0.01 atoms of He, C and N, respectively, and 0.08 additional O atoms out of total of 295 atoms, in our simulation set up at 5 GPa, 4,000 K. We have focused in our study on planets that do not accrete significant amounts of ice because the population of super-Earths and sub-Neptunes seem to be best explained with ice-free compositions^8,9,11^. However, even at 100 times solar metallicity, as in the atmosphere of Uranus^79^, the number of C, N and additional O atoms are 4, 1 and 8, respectively. Consideration of these and other additional elements, and particularly their partitioning among H-rich and H-poor phases at conditions below *T*_C_(*P*) may be important in understanding atmospheric signatures at pressure–temperature conditions far below those of our simulations^80^.

### Speciation

We define speciation using the bond criteria: two atoms *i* and *j* of type *α* and *β* are bonded if their distance *r*_*ij*_ is less than an assumed bond cutoff distance

7 $${r}_{{ij}} < {R}_{\alpha \beta }$$

We set the bond cutoff distances *R*_*αβ*_ equal to the position of the first minimum in the corresponding radial distribution function *g*_*αβ*_(*r*). We compute the radial distribution functions using the force method^81^, which yields higher precision than the more traditional binning method.

### Asymmetric regular solution model

Our phase coexistence results are captured by an asymmetric regular solution model, for which the Gibbs free energy of solution

8 $${G}_{\mathrm{sol}}=(A{x}_{1}+B{x}_{2}){x}_{1}{x}_{2}\,\left(1-\frac{T}{\tau }+\frac{P}{{\rm{\pi }}}\right)+{RT}({x}_{1}\mathrm{ln}{x}_{1}+{x}_{2}\mathrm{ln}{x}_{2})$$

where *x*_2_ = 1 − *x*_1_. The excess enthalpy (at *P* = 0)

9 $${H}_{\mathrm{ex}}=(A{x}_{1}+B{x}_{2}){x}_{1}{x}_{2}$$

The excess volume and entropy are, respectively,

10 $${V}_{\mathrm{ex}}={H}_{\mathrm{ex}}/{\rm{\pi }}$$

11 $${S}_{\mathrm{ex}}={H}_{\mathrm{ex}}/\tau $$

and *A*, *B*, *τ* and π are taken to be constants. The model is similar to a previous one^82^ except that we have included a non-zero excess volume term by generalizing their expression to permit pressure dependence of the excess Gibbs free energy. We compute two-phase equilibria numerically by setting chemical potentials of the two components in the two coexisting phases equal to each other:

12 $${\mu }_{A}^{\alpha }={\mu }_{A}^{\beta }$$

13 $${\mu }_{B}^{\alpha }={\mu }_{B}^{\beta }$$

where ${\mu }_{i}^{j}$ is the chemical potential of component *i* in phase *j*. The chemical potential of, for example, component *A* in phase *α* is

14 $${\mu }_{A}^{\alpha }={RT}\mathrm{ln}(1-{x}_{A}^{\alpha })+[B+2(A-B)(1-{x}_{A}^{\alpha })]{({x}_{A}^{\alpha })}^{2}$$

and ${x}_{A}^{j}=1-{x}_{B}^{j}$ for *j* = [*α*,* β*]. At the critical temperature^83^

15 $${\partial }^{2}{G}_{\mathrm{sol}}/\partial {x}_{1}^{2}=0$$

16 $${\partial }^{3}{G}_{\mathrm{sol}}/\partial {x}_{1}^{3}=0$$

**Extended Data Fig. 3:**
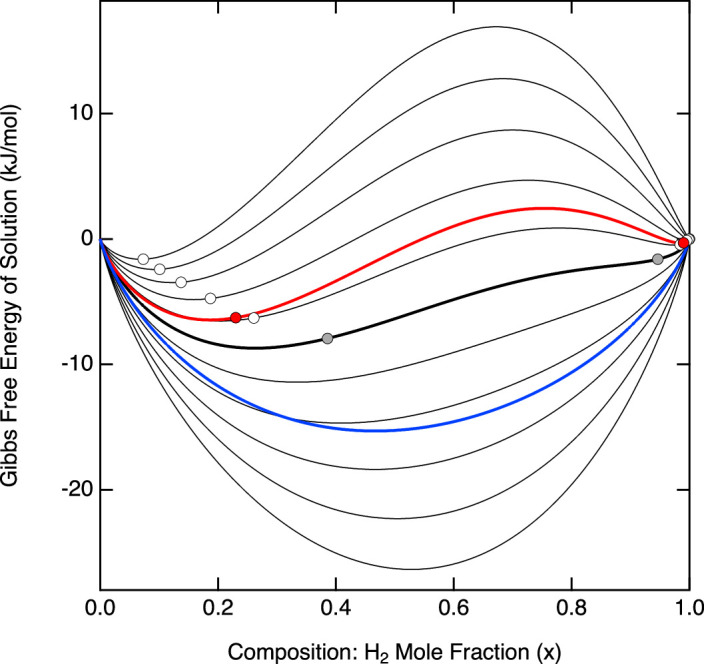
Gibbs free energy of solution. *G*_*sol*_(*x*,*T*,*P*) (equation 8) at 5 GPa (thin black lines) from 2000 K (uppermost curve) to 4000 K (lowermost curve) in increments of 200 K, and at 3000 K (bold lines) and 2 GPa (red bold), 5 GPa (black bold), 10 GPa (blue bold). Also shown for each curve corresponding to pressure, temperature conditions below the critical curve (equation 19) are the pairs of points that satisfy equations (12,13), yielding the compositions of the coexisting phases (circles).

Simultaneous solution of equations (15) and (16) yields

17 $$A=\frac{-9{x}_{{\rm{C}}}^{2}+10{x}_{{\rm{C}}}-2}{6{x}_{{\rm{C}}}^{2}{(1-{x}_{{\rm{C}}})}^{2}}\left(\frac{R{T}_{{\rm{C}}}}{1-{T}_{{\rm{C}}}/\tau +P/{\rm{\pi }}}\right)$$

18 $$B=\frac{-9{x}_{{\rm{C}}}^{2}+8{x}_{{\rm{C}}}-1}{6{x}_{{\rm{C}}}^{2}{(1-{x}_{{\rm{C}}})}^{2}}\left(\frac{R{T}_{{\rm{C}}}}{1-{T}_{{\rm{C}}}/\tau +P/{\rm{\pi }}}\right)$$

where *x*_C_ is the critical composition, and either of the equations (15) and (16) yields

19 $${T}_{{\rm{C}}}(P)={T}_{{\rm{C}}0}\left(1+\frac{P}{{\rm{\pi }}}\right)$$

where *T*_C0_ is the critical temperature at zero pressure. We find the best fitting parameter values to be *A* = 75 kJ mol^−1^, *B* = 424 kJ mol^−1^, *τ* = 4,400 K, π = −35 GPa. The Gibbs free energy of solution defined by the function (equation (8)) and its dependence on *x*, *T* and *P* is shown in Extended Data Fig. 3.

## Online content

Any methods, additional references, Nature Portfolio reporting summaries, source data, extended data, supplementary information, acknowledgements, peer review information; details of author contributions and competing interests; and statements of data and code availability are available at 10.1038/s41586-025-09970-4.

## Figures and Tables

**Extended Data Table 1:**
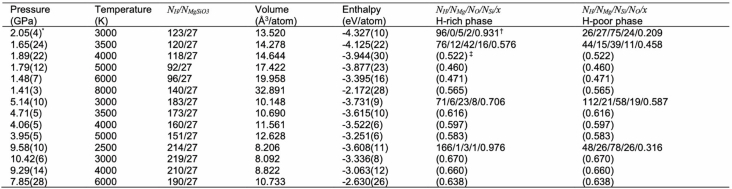
Results of phase coexistence simulations

*Uncertainties in the last digits reported in parentheses.

^†^Uncertainties in atom numbers of individual phases are a few %; uncertainties in *x* are ±0.05.

^‡^For conditions at which simulations equilibrated to a single homogeneous phase, we report the bulk value of *x* in parentheses.

**Extended Data Table 2:**
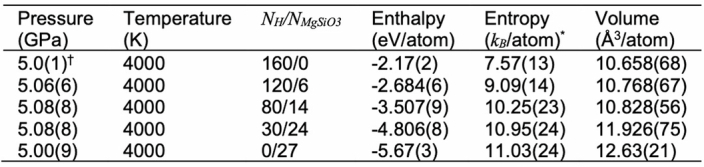
Results of homogeneous simulations

*k_*B*_ is the Boltzmann constant.

^†^Uncertainties in the last digits reported in parentheses.

## Data Availability

Data generated for this study are available in the Extended Data and at Zenodo^84^ (10.5281/zenodo.17716049).

## Code availability

All simulations are performed using the VASP code^62^.
